# Effect of Spermidine Analogues on Cell Growth of *Escherichia coli* Polyamine Requiring Mutant MA261

**DOI:** 10.1371/journal.pone.0159494

**Published:** 2016-07-19

**Authors:** Taketo Yoshida, Akihiko Sakamoto, Yusuke Terui, Koichi Takao, Yoshiaki Sugita, Kaneyoshi Yamamoto, Akira Ishihama, Kazuei Igarashi, Keiko Kashiwagi

**Affiliations:** 1 Faculty of Pharmacy, Chiba Institute of Science, 15–8 Shiomi-cho, Choshi, Chiba, 288–0025, Japan; 2 Laboratory of Bioorganic Chemistry, Department of Pharmaceutical Technology, Josai University, 1–1 Keyaki-dai, Sakado, Saitama, 350–0295, Japan; 3 Department of Frontier Bioscience, Hosei University, Koganei, Tokyo, 184–8584, Japan; 4 Amine Pharma Research Institute, Innovation Plaza at Chiba University, 1-8-15, Inohana, Chuo-ku, Chiba, Chiba 260–0856, Japan; 5 Graduate School of Pharmaceutical Sciences, Chiba University, 1-8-1 Inohana, Chuo-ku, Chiba, Chiba, 260–8675, Japan; University of Quebec at Trois-Rivieres, CANADA

## Abstract

The effects of spermidine analogues [norspermidine (NSPD, 33), spermidine (SPD, 34), homospermidine (HSPD, 44) and aminopropylcadaverine (APCAD, 35)] on cell growth were studied using *Escherichia coli* polyamine-requiring mutant MA261. Cell growth was compared at 32°C, 37°C, and 42°C. All four analogues were taken up mainly by the PotABCD spermidine-preferential uptake system. The degree of stimulation of cell growth at 32°C and 37°C was NSPD ≥ SPD ≥ HSPD > APCAD, and SPD ≥ HSPD ≥ NSPD > APCAD, respectively. However, at 42°C, it was HSPD » SPD > NSPD > APCAD. One reason for this is HSPD was taken up effectively compared with other triamines. In addition, since natural polyamines (triamines and teteraamines) interact mainly with RNA, and the structure of RNA is more flexible at higher temperatures, HSPD probably stabilized RNA more tightly at 42°C. We have thus far found that 20 kinds of protein syntheses are stimulated by polyamines at the translational level. Among them, synthesis of OppA, RpoE and StpA was more strongly stimulated by HSPD at 42°C than at 37°C. Stabilization of the initiation region of *oppA* and *rpoE* mRNA was tighter by HSPD at 42°C than 37°C determined by circular dichroism (CD). The degree of polyamine stimulation of OppA, RpoE and StpA synthesis by NSPD, SPD and APCAD was smaller than that by HSPD at 42°C. Thus, the degree of stimulation of cell growth by spermidine analogues at the different temperatures is dependent on the stimulation of protein synthesis by some components of the polyamine modulon.

## Introduction

Polyamines [putrescine (PUT), spermidine (SPD) and spermine (SPM)] are present at millimolar concentrations in both prokaryotic and eukaryotic cells, and play important roles in cell proliferation and viability [1–4]. To study physiological functions of polyamines, we estimated polyamine distribution in cells, and found that polyamines exist mostly as polyamine-RNA complexes [5, 6], and thus affect translation at various steps [7]. In *E*. *coli*, wild type W3110 (A-type) [8], the amount of SPD bound to DNA and RNA was estimated to be 0.36 and 1.4, and that of PUT was 3.1 and 3.5 mol/100 mol phosphates of DNA and RNA, respectively [6, 7]. Polyamines enhance total protein synthesis through stimulation of the assembly of 30S ribosomal subunits [9], and also the addition of polyamines stimulates the synthesis of some specific proteins, mainly transcription factors [10], in *E*. *coli* MA261 cells which cannot synthesize polyamines [11]. Genes encoding proteins regulated by polyamines at the level of translation were termed the “polyamine modulon” [2], and thus far 20 members of the polyamine modulon have been identified [7, 12]. The mechanism of stimulation of protein synthesis involving genes in the polyamine modulon is as follows: 1) Polyamines stimulate protein synthesis when a Shine-Dalgarno (SD) sequence in the mRNA is distant from the initiation codon AUG; 2) Polyamines stimulate protein synthesis when the inefficient initiation codon UUG or GUG is present in the mRNA; and 3) Polyamines stimulate protein synthesis when the open reading frame in mRNAs contains a termination codon. In this case, polyamines stimulate suppressor tRNA binding or +1 frameshift [7]. For polyamine effects at the level of DNA, it has been reported that polyamines can stimulate B to Z conversion of poly(dG-m^5^dC)•poly(dG-m^5^dC) in a cell-free systems [13, 14], but thus far there is no report using intact cells.

In *E*. *coli*, the major triamine is SPD [2], but norspermidine (NSPD) and homospermidine (HSPD) are synthesized in some bacteria such as *Vibrio alginolyticus* [15], *Acinetobacter tartarogenes* [16] and extreme thermophiles [17, 18]. Furthermore, growth of the *E*. *coli* polyamine requiring mutant DR112 in which APCAD can be synthesized, was faster than that of another polyamine requiring mutant MA261, in which APCAD cannot be synthesized in the absence of polyamines [19, 20]. Thus, we studied the effect of four triamines on cell growth using the *E*. *coli* polyamine requiring mutant MA261 at different temperatures 32°C, 37°C and 42°C. The results indicate that the triamine most effective for cell growth is different at different temperatures. One reason for that is probably due to a difference in maintenance of a suitable RNA structure by interaction of different triamines at different temperatures.

## Materials and Methods

### Bacterial Strains and Culture Conditions

A polyamine-requiring mutant of *E*. *coli* MA261 (*speB speC gly leu thr thi*) [11] was kindly supplied by Dr. Maas. Then, its spermidine uptake deficient mutant MA261 *potD*::*Km*, and its putrescine and spermidine uptake deficient mutant KK3131, were prepared as described previously [21, 22]. These three kinds of *E*. *coli* strains were cultured overnight at 37°C in modified Luria-Bertani (LB) medium (10 g of tryptone, 5 g of yeast extract and 5 g of NaCl per liter). Eighty μl of bacterial culture were transferred to 10 ml of medium A (40.2 mM K_2_HPO_4_, 22.1 mM KH_2_PO_4_, 1.7 mM sodium citrate, 7.6 mM (NH_4_)_2_SO_4_, 0.41 mM MgSO_4_, 6 μM thiamine, 40 μM biotin, 0.8 mM leucine, 0.8 mM threonine, 0.7 mM methionine, 1 mM serine, 1 mM glycine, 0.6 mM ornithine, pH 6.8) with 0.4% glucose (22.4 mM), and *E*. *coli* cells were grown till A_540_ reached 1.0. Then, *E*. *coli* cells were cultured at an A_540_ of 0.1 and growth was monitored at 32°C, 37°C or 42°C by measuring A_540_ in medium A containing 0.4% glucose with or without 0.1 mM triamine (NSPD, SPD, HSPD or APCAD).

### Triamines

Norspermidine (NSPD, 33) and spermidine (SPD, 34) were purchased from Wako Pure Chem. (Tokyo, Japan). Homospermidine (HSPD, 44) and aminopropylcadaverine (APCAD, 35) were synthesized according to the methods published by Niitsu et al. [23, 24].

### Western Blot Analysis

*E*. *coli* cells were harvested at A_540_ = 0.3, and cell lysate was prepared as described previously [20]. Protein content was determined by the method of Bradford [25]. Western blot analysis was performed by the method of Nielsen et al. [26], using ECL Western blotting reagents (GE Healthcare Bio-Sciences) using 10 μg protein. Antibodies against OppA, RpoE (σ^24^), StpA, EmrR and RpoD (σ^70^) were prepared by injecting 1 mg each of StpA, RpoE (σ^24^), OppA and RpoD (σ^70^) with Freund’s complete adjuvant to a rabbit [27]. The level of protein on the blot was quantified with a LAS-3000 luminescent image analyzer (Fuji Film).

### Measurement of Polyamine Contents

Polyamines in *E*. *coli* were measured by the method published before [20] with some modifications using 5% trichloroacetic acid (TCA) supernatant and tandemly connected double columns of TSKgel Polyaminepak (4.6 x 50 mm) (Tosoh) heated to 50°C. The flow rate of the buffer (0.35 M citric acid buffer, pH 5.35, 2 M NaCl and 20% methanol) was 0.20 ml/min. Detection of polyamines was by fluorescence intensity after reaction of the column effluent at 50°C with *o*-phthalaldehyde solution. The retention times for NSPD, SPD, HSPD and APCAD were 42.5, 47.2, 50.6 and 67.7 min, respectively.

### Circular Dichroism (CD) Measurement

CD spectra of RNAs were recorded over 200–320 nm on a Jasco J-820 spectropolarimeter using a 0.1-cm path length cuvette at 37°C or 42°C [28]. Scan speed was 100 nm/min, and CD samples contained 10 mM Tris-HCl, pH 7.5, 50 mM KCl and 50 μM RNA. Where indicated, a specified concentration of MgCl_2_ or a triamine with 1 mM Mg^2+^ was added to the CD samples. The *K*_d_ values were determined according to the double reciprocal equation plot using concentration-dependent shift values in elliplicity induced by triamines or Mg^2+^ at 208 nm.

### Measurement of Dissociation Constant (*K*_d_) of Triamines for PotD Protein

PotD protein, a substrate binding protein of spermidine-preferential uptake system, i.e. PotABCD uptake system [29], was purified as described previously [21]. The reaction mixture (0.1 ml) contained 10 mM Tris-HCl (pH7.5), 50 mM KCl, 5 μM (195 μg/ml) PotD protein and various concentrations (0, 2, 4, 6, 8, 10, 20 and 40 μM) of triamines. CD spectra were obtained as described above except that CD spectra of PotD were recorded over 200–250 nm and scan speed was 50 nm/min. The *K*_d_ values of triamines for PotD protein were determined by the shift of elliplicity at 208 nm according to the double reciprocal equation plot.

### Statistics

Values are indicated as mean ± S. E. of triplicate determinations. Data of control and treated groups were analyzed by Student’s *t* test, and a statistical difference was shown by probability values.

## Results

### Effect of Triamines on Cell Growth of a Polyamine Requiring Mutant MA261 at 32°C, 37°C and 42°C

**Fig 1:**
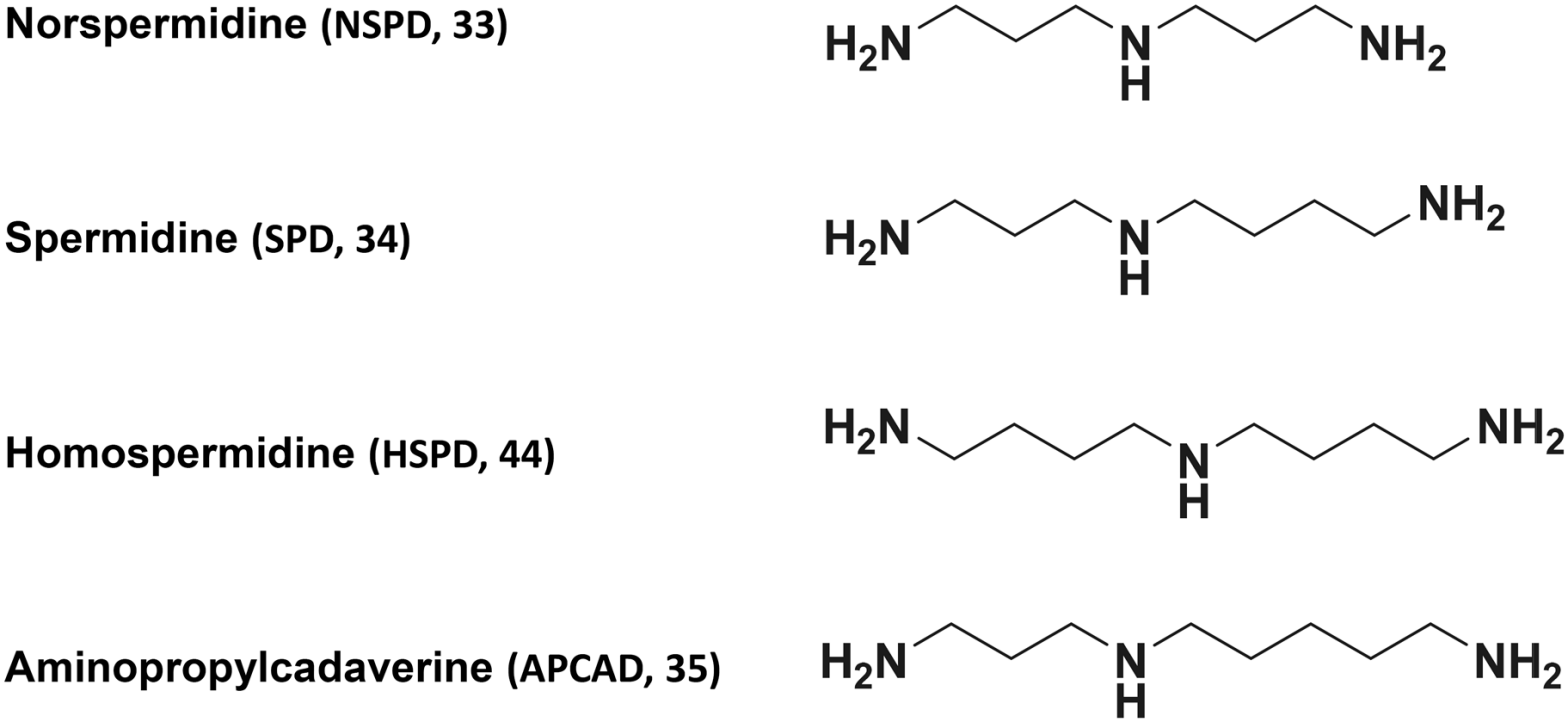
Structure of triamines.

**Fig 2:**
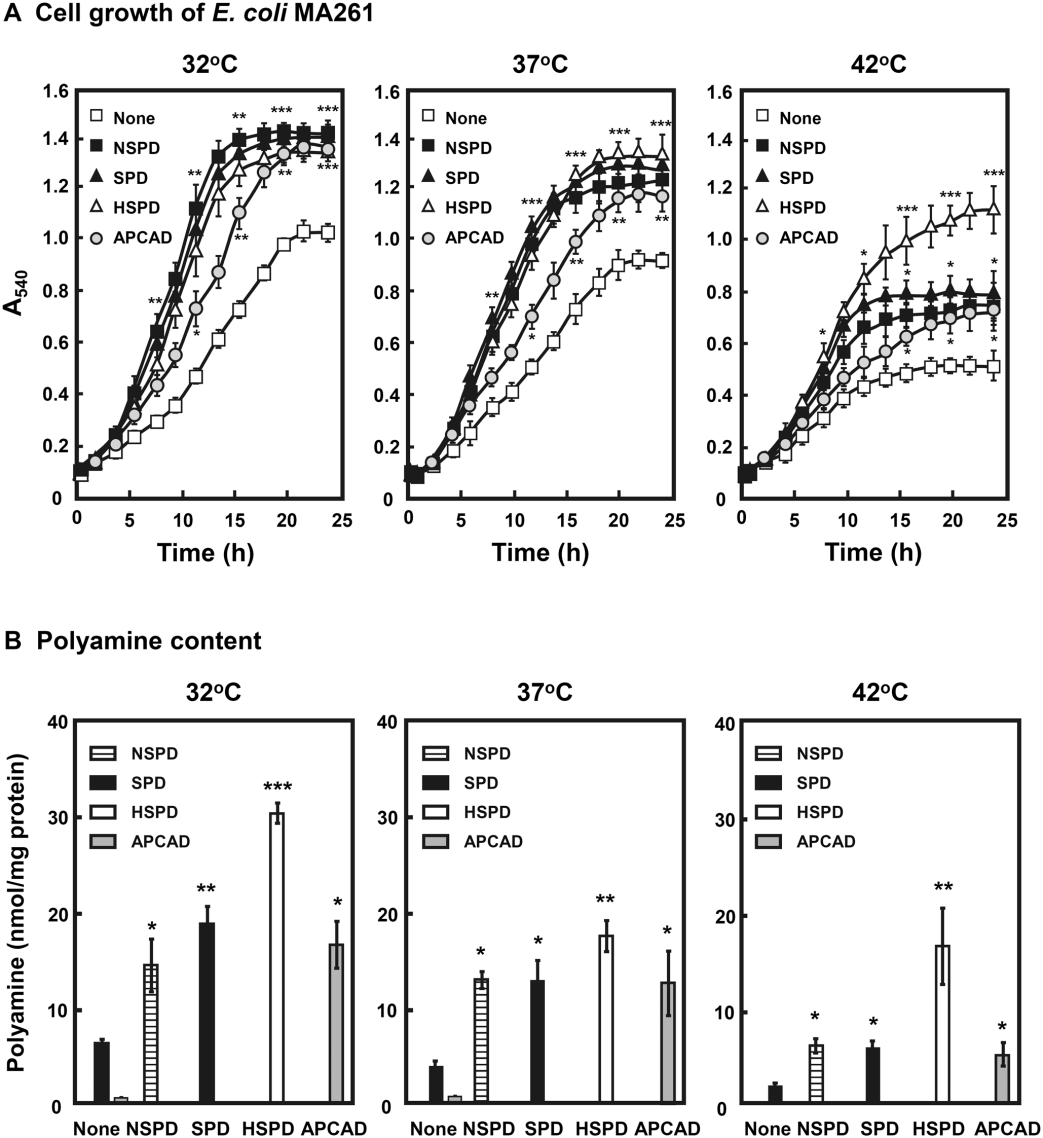
Effect of triamines on growth of *E*. *coli* MA261. A. Growth of *E*. *coli* MA261. Cell growth at 32°C, 37°C and 42°C in the presence and absence of 0.1 mM triamines was followed by measuring A_540_. B. Polyamine content in cells at A_540_ = 0.3 was measured as described in Materials and Methods. Values are mean ± S. E. of triplicate determinations. Student’s t test was performed for the value obtained in the presence and absence of triamine. * *p* < 0.05; ** *p* < 0.01; *** *p* < 0.001. The growth of *E*. *coli* MA261 in the presence of triamine was significantly faster than that in the absence of triamine.

The effects of NSPD, SPD, HSPD and APCAD (Fig 1) on growth of the *E*. *coli* polyamine requiring mutant MA261 were compared at 32°C, 37°C and 42°C (Fig 2A). The growth of *E*. *coli* MA261 was stimulated greatly by 0.1 mM each of NSPD, SPD and HSPD at almost the same extent, and less effectively by APCAD compared to the absence of triamines at both 32°C and 37°C. At 42°C, growth of MA261 was slower than growth at 32°C and 37°C, and was most effectively stimulated by HSPD (Fig 2A). The growth of *E*. *coli* wild type W3110 (A-type) [8] was not influenced by 0.1 mM each of NSPD, SPD, HSPD and APCAD (data not shown).

Levels of polyamines in cells were measured by HPLC using TCA soluble extract of cells (Fig 2B). When *E*. *coli* MA261 cells were cultured without triamines, only small amount of SPD was detected in the cells. The addition of a given triamine to the medium increased its content in cells at 32°C, 37°C, and 42°C, and the level of HSPD was highest among the triamines. At 42°C, levels of NSPD, SPD and APCAD were much lower compared to cells cultured at 32°C and 37°C, whereas level of HSPD was similar at 37°C and 42°C. These results suggest that a high level of HSPD in cells supports the enhanced growth of MA261 at 42°C. The content of APCAD in cells was nearly equal to that of NSPD and SPD, but the stimulatory effect on cell growth was less than that seen with NSPD and SPD, perhaps because the interaction between APCAD and RNA is weak compared to NSPD and SPD.

**Fig 3:**
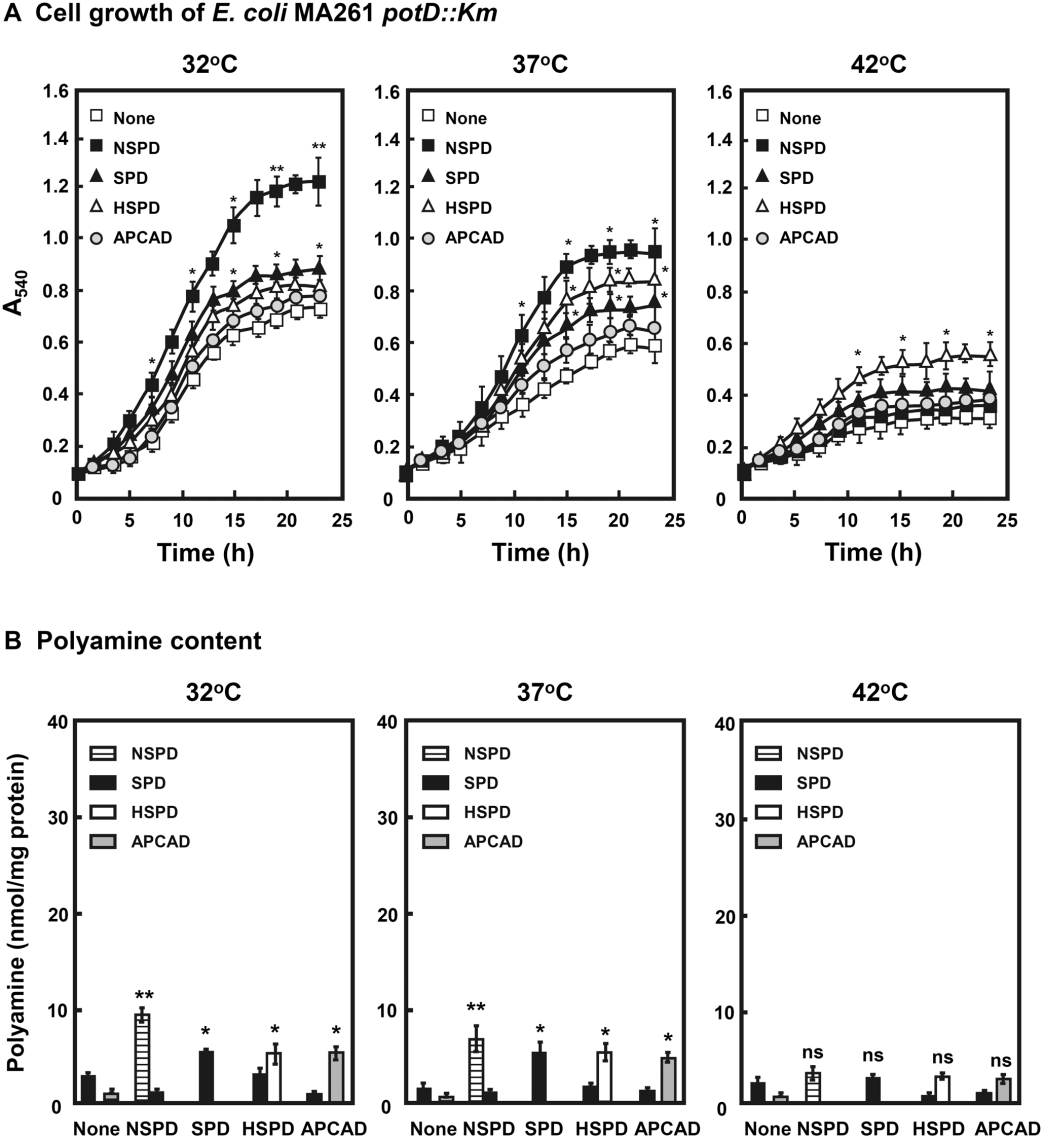
Effect of triamines on cell growth of *E*. *coli* MA261 *potD*::*Km*. A and B. Cell growth of *E*. *coli* MA261 *potD*::*Km* and polyamine content were measured as described in the legend of Fig 2. ns, *p* ≥ 0.05; * *p* < 0.05; ** *p* < 0.01.

It has been reported that the PotABCD transporter, an ATP binding cassette transporter, catalyzes the uptake of spermidine [30]. Thus, we tested whether all triamines are taken up by the PotABCD transport system. Among four proteins in PotABCD, PotD protein is the substrate binding protein [31, 32]. So, the effects of triamines on cell growth were studied using a PotD-deficient mutant of MA261 (MA261 *potD*::*Km*) [21]. As shown in Fig 3A, stimulatory effects on growth of MA261 *potD*::*Km* by triamines decreased greatly compared with that of cell growth of MA261 at all temperature tested (32°C, 37°C and 42°C). Since NSPD stimulated growth of MA261 *potD*::*Km* relatively well compared with other triamines at 32°C, NSPD may be taken up by another transport system in addition to PotABCD SPD uptake system at 32°C. Similar results were obtained with *E*. *coli* KK3131 which is deficient in both putrescine and spermidine uptake [22] (data not shown). The content of triamines in MA261 *potD*::*Km* cells was also determined (Fig 3B). Triamine content was very low compared with MA261 cells, indicating that triamines are mainly taken up by the PotABCD transport system. NSPD content in MA261 *potD*::*Km* cells was highest among triamines at 32°C and 37°C.

**Fig 4:**
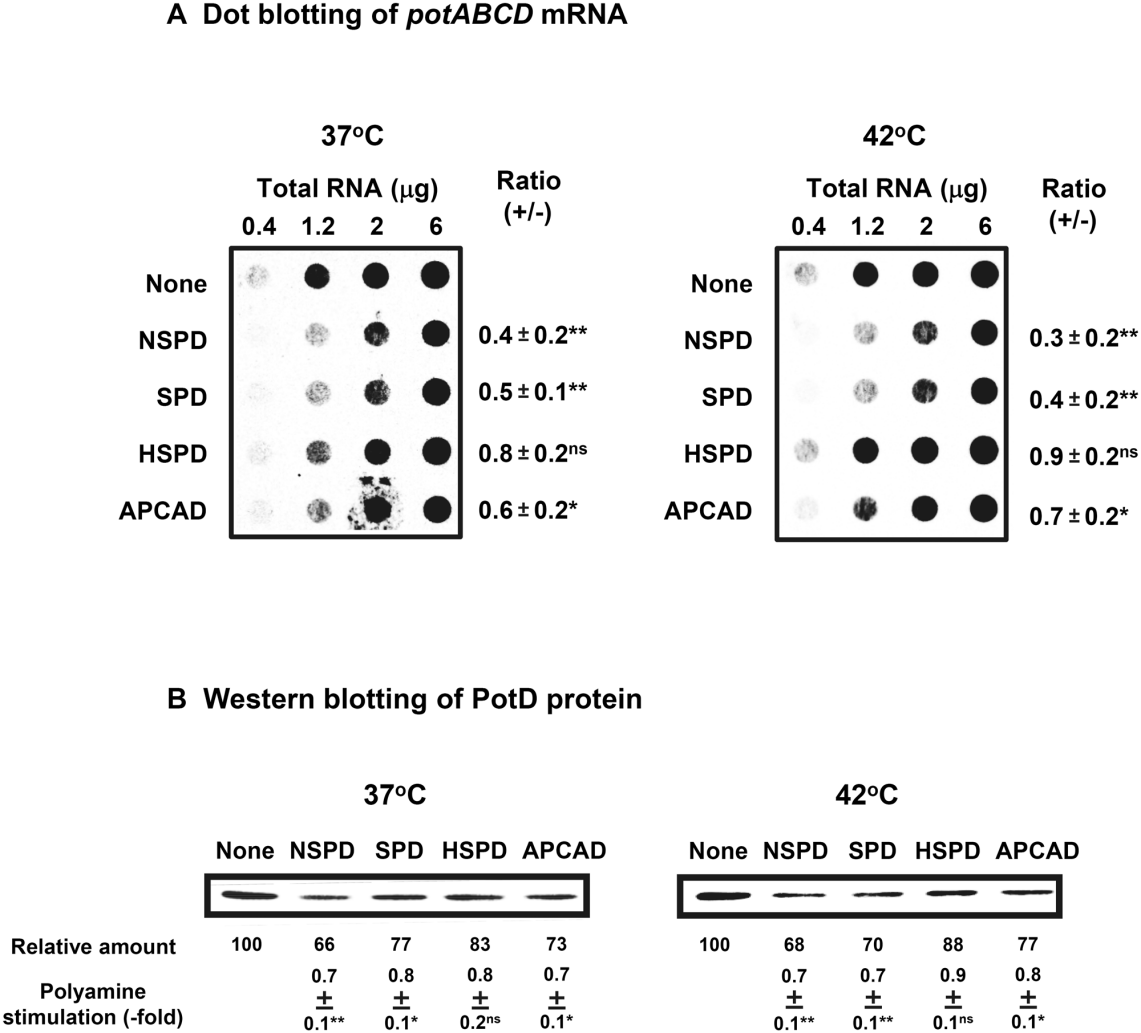
Levels of *potABCD* mRNA and PotD protein in *E*. *coli* MA261 cultured in the presence and absence of triamines. Cells were harvested at A_540_ = 0.3. A. The level of *potABCD* mRNA was measured by dot blotting [12]. The probe for dot blotting was prepared by PCR using primers, 5’-CGACTTACGAGTCGAACGAAACCATGTACG-3’ and 5’-AGAACCGTTCCAGATCATGCCGAGGTTAAC-3’. B. The level of PotD protein was measured by Western blotting using 10 μg protein. Values are mean ± S. E. of triplicate determinations. ns, *p* ≥ 0.05; *, *p* < 0.05; **, *p* < 0.01.

Why HSPD is taken up into cells effectively, especially at 42°C was investigated. We have previously shown that the SPD-PotD complex inhibits transcription of *potABCD* operon [33]. Thus, the affinity of triamines for PotD was first compared. As shown in Table 1, the affinity for PotD was in the order NSPD > SPD > APCAD > HSPD at both 37°C and 42°C. Accordingly the degree of inhibition of the transcription of *potABCD* operon was in the order NSPD > SPD > APCAD > HSPD (Fig 4A). The level of PotD protein was parallel with that of *potABCD* mRNA, and was highest when cells were cultured with HSPD (Fig 4B). These results suggest that the strongest stimulation of cell growth by HSPD among four triamines at 42°C was mainly due to the most effective uptake of HSPD, because the level of PotABCD transporter was the highest in the presence of HSPD.

**Table 1:** Affinity of triamines for PotD protein.

Polyamine	*K*_d_ (μM)	
	37°C	42°C
NSPD	2.4 ± 0.1	3.7 ± 0.2
SPD	3.2 ± 0.1	5.4 ± 0.1
HSPD	8.8 ± 0.4	13.0 ± 0.2
APCAD	5.8 ± 0.2	8.0 ± 0.4

The *K*_d_ values were determined as described in Materials and Methods.

### Effects of Triamines on Various Kinds of Protein Synthesis and Structural Changes of Their mRNAs at 37°C and 42°C

**Fig 5:**
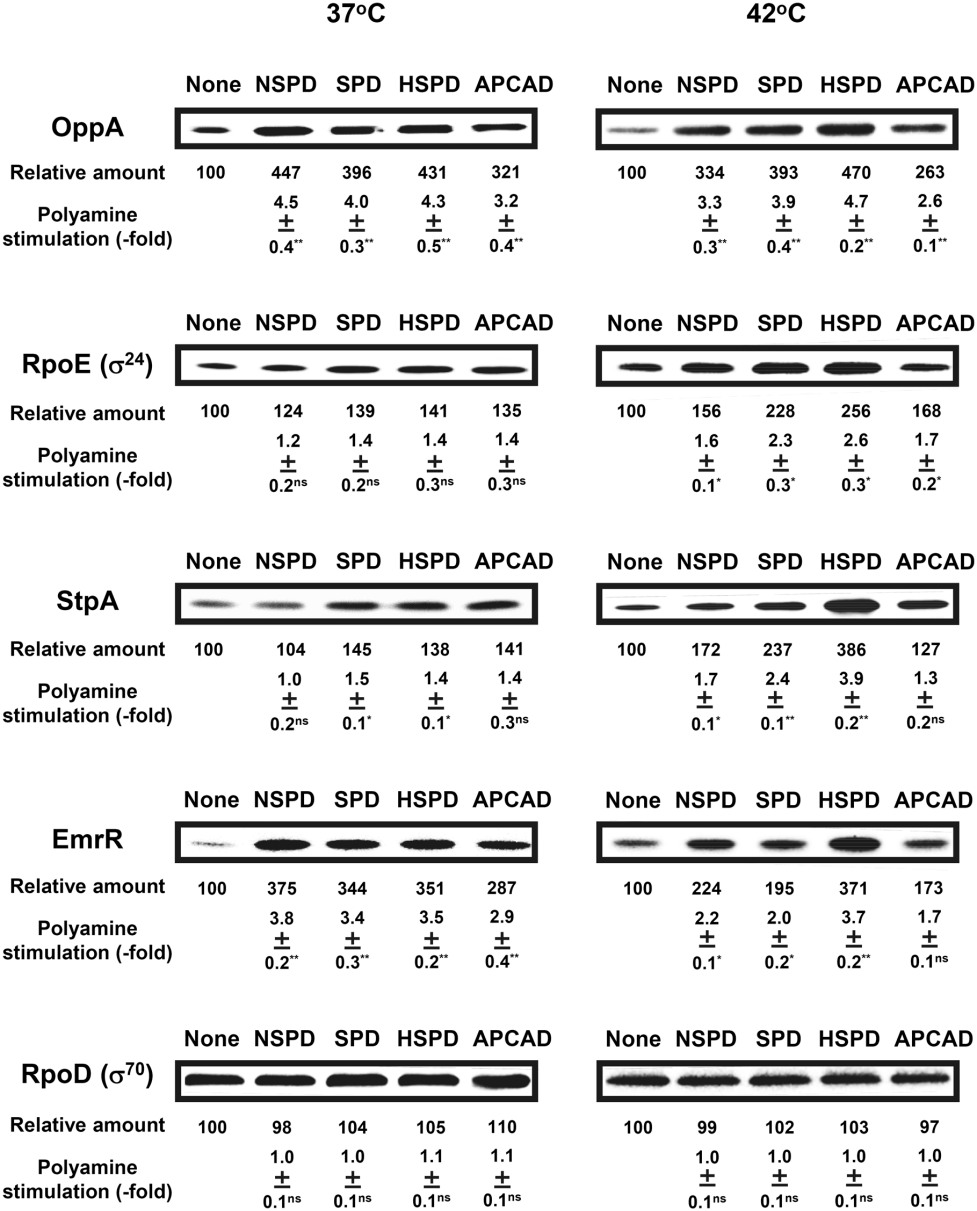
Levels of various proteins encoded by the polyamine modulon in *E*. *coli* MA261 cultured in the presence and absence of triamines. The levels of OppA, RpoE (σ^24^), StpA and EmrR were measured together with a control protein, RpoD (σ^70^) by Western blotting using 10 μg protein. Values are mean ± S. E. of triplicate determinations. The degree of stimulation of protein synthesis by triamines was measured using a Las-3000 luminescent image analyzer. ns, *p* ≥ 0.05; * *p* < 0.05; ** *p* < 0.01.

We have previously shown that polyamines stimulate several kinds of protein syntheses which are important for cell growth and viability [2, 7]. These effects were caused by structural change of mRNAs [7, 12]. As shown in Fig 5, synthesis of OppA (oligopeptide binding protein), RpoE (σ^24^ transcription factor for heat shock response genes), StpA (transcription factor for the genes of flagellin and ribosomal proteins) and EmrR (negative transcription factor for the genes of drug excretion proteins) were stimulated by NSPD, SPD, HSPD and APCAD to a similar degree at 37°C, except for a lack of effect of NSPD on StpA synthesis. As a control, effects of triamines on RpoD (σ^70^ transcription factor, a major σ factor) synthesis were measured, and triamines did not stimulate RpoD synthesis significantly.

A different pattern of effects was seen at 42°C (Fig 5). The degree of stimulation by HSPD of OppA, RpoE, StpA and EmrR synthesis was largest among the four triamines. In the control, RpoD synthesis was not affected. These results suggest that the structure of RNA is more flexible at 42°C than at 37°C, and HSPD maintains an active RNA structure more effectively than the other three triamines.

**Fig 6:**
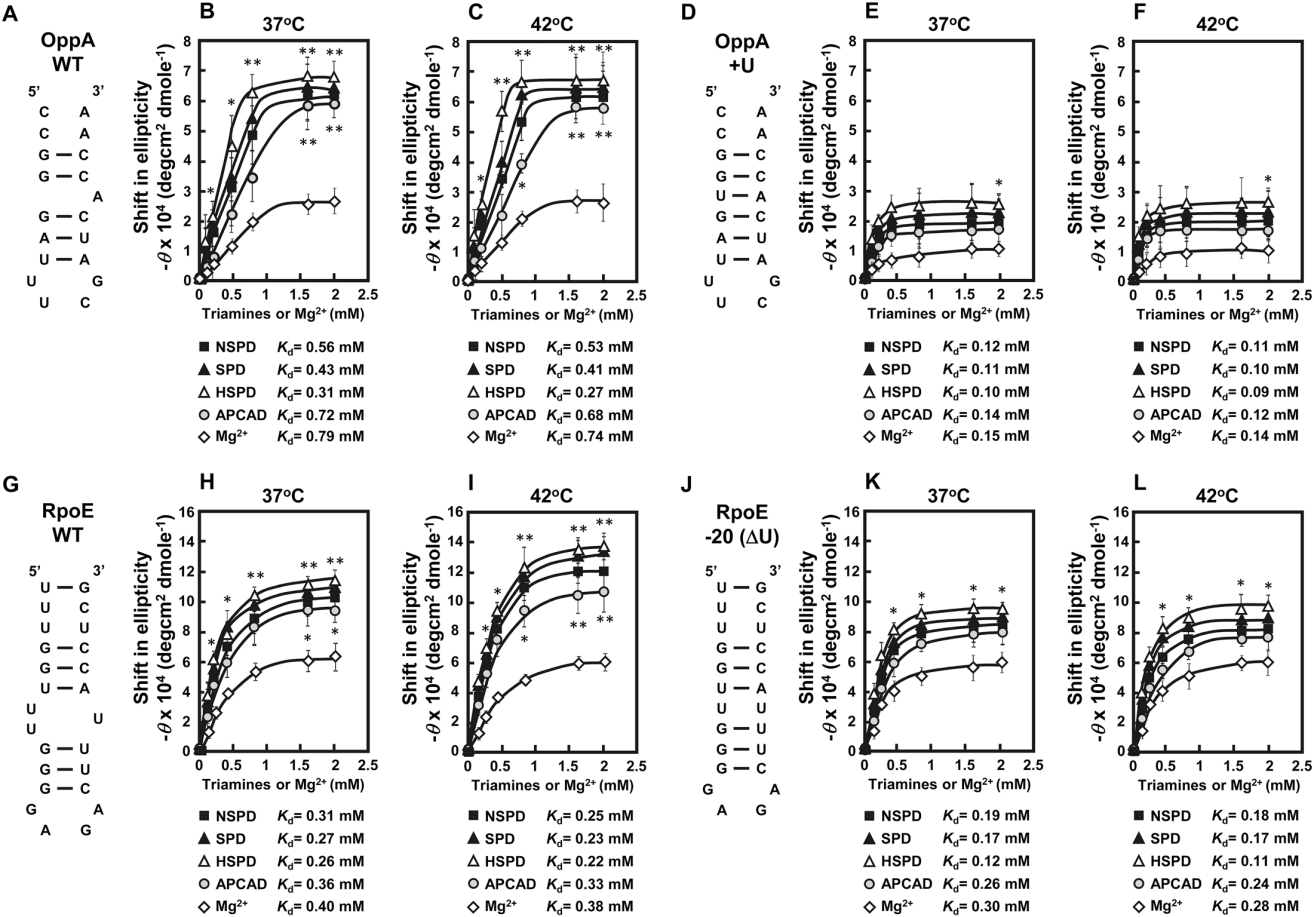
Stabilization of OppA and RpoE WT RNAs, and more stable modified RNAs measured by CD. A, D, G and J. Structures of OppA WT RNA, OppA + U RNA, RpoE WT RNA, and RpoE -20(ΔU) RNA are shown. B and C (OppA WT RNA), E and F (OppA + U RNA), H and I (RpoE WT RNA), and K and L (RpoE -20(ΔU) RNA). Concentration-dependent shifts induced by Mg^2+^ or a triamine with 1 mM Mg^2+^ at 37°C and 42°C in elliplicity at 208 nm are shown. Values are mean ± S. E. of triplicate determinations. The *K*_d_ values of Mg^2+^ and a triamine with 1 mM Mg^2+^ were determined by the double reciprocal equation plot.

The structural change of the initiation region of OppA and RpoE mRNAs by triamines was evaluated by CD. As a control, CD spectra of more stable RNAs [OppA + U and RpoE -20(ΔU)], which lacks bulged-out region, were measured. CD spectra of wild type OppA RNA, OppA + U RNA, wild type RpoE RNA, and RpoE -20(ΔU) RNA at 42°C were shown in S1 Fig. A substantial increase in the negative band at 208 nm in CD reflects stabilization (or an increase) of the A-form double stranded RNA [28], and was measured as a shift of elliplicity at 208 nm (Fig 6). In addition, dissociation constant (*K*_d_) of triamines for the initiation region of OppA and RpoE mRNAs was measured after changing the concentrations of Mg^2+^ or a triamine in the presence of 1 mM Mg^2+^. We have previously shown a structural change of the bulged-out region of double-stranded RNA induced by spermidine is strongly involved in polyamine stimulation of specific kinds of protein syntheses [12, 34]. As shown in Fig 6, stabilization of the bulged-out region of double-stranded RNA existing close to the initiation codon AUG of both OppA and RpoE mRNAs was in the order HSPD > SPD > NSPD > APCAD at both 37°C and 42°C (Fig 6A~6C and 6G~6I). Stabilization by triamines was similar in both 19-mer OppA RNA and 25-mer RpoE RNA. The degree of stabilization of both RNAs by triamines was more significant at 42°C than at 37°C judging from their *K*_d_ values. Stabilization of more stable RNAs [OppA + U and RpoE -20(ΔU)] by triamines (Fig 6D~6F and 6J~6L) was less than wild type RNAs at both 37°C and 42°C, because these RNAs were more stable than wild type RNAs in the presence of Mg^2+^ only. These findings support the results shown in Fig 2, where HSPD stimulates cell growth more effectively at 42°C.

## Discussion

Our results indicate that the degree of the stimulation of cell growth of *E*. *coli* MA261 by four triamines is different at 32°C, 37°C and 42°C. This is probably due to the difference of the ability of triamines to cause the structural change of RNA and that of the uptake activities of triamines at different temperatures. In the latter case, it is noted that HSPD is effectively taken up at 42°C, because the inhibition of the transcription of *potABCD* operon by PotD-triamine complex was the weakest in PotD-HSPD (Fig 4). As for the interaction of triamine with RNA, the stabilization of RNA by Mg^2+^ is primarily taken into consideration. However, Mg^2+^ is not sufficient to maintain a fully active structure of RNA. We have previously found that polyamines not only lower the optimal Mg^2+^ concentration, but also stimulate protein synthesis in *E*. *coli* and rat liver cell free systems, and that the optimal concentration of polyamines was in the order putrescine > spermidine > spermine [35].

Since the structure of RNA is flexible, we have determined how it is affected by four triamines (NSPD, SPD, HSPD and APCAD) at different temperatures (32°C, 37°C and 42°C) by measuring effect of triamines on cell growth and protein synthesis [2, 7]. The results indicate that a short triamine, NSPD, was most effective at 32°C and a long triamine, HSPD, was most effective at 42°C. These results strongly suggest that HSPD is best able to stabilize flexible RNA at 42°C, and NSPD is best able to stabilize relatively inflexible RNA at 32°C. APCAD was the least effective triamine at all temperatures studied, suggesting that a distance of five carbons between two amino groups (as found in APCAD) is not optimal for interaction with phosphate groups in RNA, compared to the 4-carbon spaced in SPD and HSPD. The average distance between phosphate groups of A-form RNA (protein Data Bank code 1570) is 6.35 Å for the major groove and 5.20 Å for the minor groove. These are similar to the N-N distances of the diaminobutane and diaminopropane moieties in all-trans spermidine, which are ~6.2 and 5.0 Å, respectively [36]. The N-N distance of diaminopentane (~7.4 Å), found in APCAD, may be too long to effectively interact with the phosphate group in RNA even at 42°C.

We have previously shown that effects of polyamines are mainly caused by stabilization of a bulged-out region of double-stranded RNA, whose structure is not stabilized by Mg^2+^. The degree of SPD stimulation of OppA and RpoE synthesis through stabilization of the bulged-out region of double-stranded RNA at 37°C was 5.1- and 2.3-fold, respectively, when MA261 cells were cultured in the presence of putrescine and harvested at A_540_ = 0.5 [34, 37]. In this case, transported putrescine can be converted to SPD and both putrescine and SPD existed in cells. In this study, cells were cultured in the presence and absence of a triamine and harvested at A_540_ = 0.3. So, the degree of polyamine stimulation was slightly different from that reported in previous studies [34, 37].

Many bacteria probably adapt to the environmental temperature through synthesizing suitable polyamines to interact with, and stabilize RNA. For example, *Thermus thermophiles* can grow at 78°C in the presence of long and branched polyamines [17]. It would be of interest to study the structure and flexibility of RNAs at that temperature, and the molecular interactions with long and branched polyamines.

## Supporting Information

S1 Fig CD spectra of OppA and RpoE WT RNAs, and more stable modified RNAs at 42°C. CD spectra were recorded as described in Materials and Methods.
(PPTX)
