# Supplementary material for: Effect of Spermidine Analogues on Cell Growth of Escherichia coli Polyamine Requiring Mutant MA261
Source: PLoS One. 2016 Jul 19;11(7):e0159494. doi: 10.1371/journal.pone.0159494 (PMC4951125; doi:10.1371/journal.pone.0159494)
Supplement: S1 Fig — CD spectra were recorded as described in Materials and Methods. (PPTX) [file pone.0159494.s001.pptx]

## Slide 1
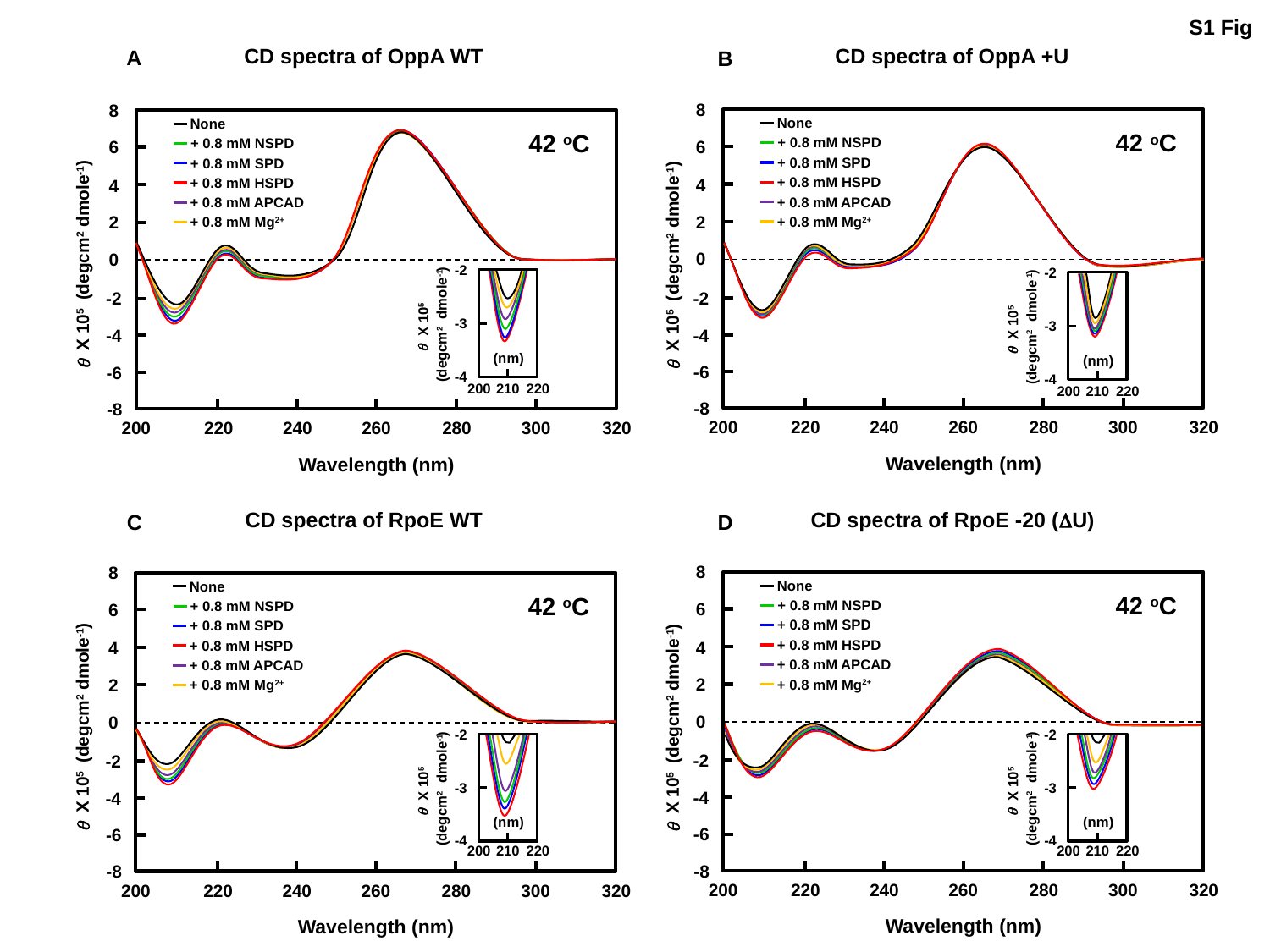

S1 Fig
CD spectra of OppA WT
CD spectra of OppA +U
A
B
8
8
None
+ 0.8 mM NSPD
+ 0.8 mM SPD
+ 0.8 mM HSPD
+ 0.8 mM APCAD
+ 0.8 mM Mg2+
None
+ 0.8 mM NSPD
+ 0.8 mM SPD
+ 0.8 mM HSPD
+ 0.8 mM APCAD
+ 0.8 mM Mg2+
42 oC
42 oC
6
6
4
4
2
2
 (degcm2 dmole-1)
 (degcm2 dmole-1)
0
0
-2
)
　dmole-1
 (degcm2
 X 105
q
-3
(nm)
-4
200
210
220
-2
)
　dmole-1
 (degcm2
 X 105
q
-3
(nm)
-4
200
210
220
-2
-2
-4
-4
 X 105
 X 105
q
-6
q
-6
-8
-8
200
220
240
260
280
300
320
200
220
240
260
280
300
320
Wavelength (nm)
Wavelength (nm)
CD spectra of RpoE WT
CD spectra of RpoE -20 (DU)
C
D
8
8
None
+ 0.8 mM NSPD
+ 0.8 mM SPD
+ 0.8 mM HSPD
+ 0.8 mM APCAD
+ 0.8 mM Mg2+
None
+ 0.8 mM NSPD
+ 0.8 mM SPD
+ 0.8 mM HSPD
+ 0.8 mM APCAD
+ 0.8 mM Mg2+
42 oC
42 oC
6
6
4
4
2
2
 (degcm2 dmole-1)
 (degcm2 dmole-1)
0
0
-2
)
　dmole-1
 (degcm2
 X 105
q
-3
(nm)
-4
200
210
220
-2
)
　dmole-1
 (degcm2
 X 105
q
-3
(nm)
-4
200
210
220
-2
-2
-4
-4
 X 105
 X 105
q
-6
q
-6
-8
-8
200
220
240
260
280
300
320
200
220
240
260
280
300
320
Wavelength (nm)
Wavelength (nm)
